# Estimation of genetic components, heterosis and combining ability of elite Pakistani wheat varieties for yield attributing traits and stripe rust response

**DOI:** 10.18699/VJGB-23-72

**Published:** 2023-10

**Authors:** M.S. Ahmed, M. Qamar, S. Waqar, A. Naeem, R.A. Javaid, S.K. Tanveer, I. Hussain

**Affiliations:** Wheat Program, Crop Sciences Institute, National Agricultural Research Center, Islamabad, Pakistan Rice Program, Crop Sciences Institute, National Agricultural Research Center, Islamabad, Pakistan; Wheat Program, Crop Sciences Institute, National Agricultural Research Center, Islamabad, Pakistan; Wheat Program, Crop Sciences Institute, National Agricultural Research Center, Islamabad, Pakistan; Rice Program, Crop Sciences Institute, National Agricultural Research Center, Islamabad, Pakistan; Wheat Program, Crop Sciences Institute, National Agricultural Research Center, Islamabad, Pakistan; Wheat Program, Crop Sciences Institute, National Agricultural Research Center, Islamabad, Pakistan; Wheat Program, Crop Sciences Institute, National Agricultural Research Center, Islamabad, Pakistan

**Keywords:** wheat, combining ability, mid-parent heterosis, stripe rust, rust resistance index, пшеница, комбинационная способность, гетерозис, желтая ржавчина злаков, индекс устойчивости к ржавчине

## Abstract

Wheat (Triticum aestivum L.) is a staple food and major source of dietary calories in Pakistan. Improving wheat varieties with higher grain yield and disease resistance is a prime objective. The knowledge of genetic behaviour of germplasm is key. To achieve this objective, elite wheat varieties were crossed in 4 by 3, line × tester design, and tested in 2019 in a triplicate yield trial to estimate genetic variance, general and specific combining ability, mid-parent heterosis and stripe rust (Puccinia striiformis L.). High grain 3358 kg·ha–1 was recorded in F1 hybrid (ZRG-79 × PAK-13). Analysis of variance (ANOVA) revealed significant genotypic variance in grain yield. Broad sense heritability (H2) was recorded in the range of 28 to 100 %. General combining ability (GCA) significant for grain yield in parents except FSD-08 and PS-05 was recorded, while specific combining ability (SCA) was recorded to be highly significant for grain yield only in two crosses (ZRG-79 × NR-09 and ZRG-79 × PAK-13). Mid-parent heterosis was estimated in the range of –28 to 62.6 %. Cross combinations ZRG-79 × PAK-13 depicted highly significant mid-parent heterosis (62.6 %). Highly significant correlation was observed among spike length, spikelets per spike, plant height and 1000-grain weight. Rust resistance index was recorded in the range of 0 to 8.5. These findings suggest exploitation of GCA for higher grain yield is important due to the presence of additive gene action and selection in the filial generations will be effective with improved rust resistance, while cross combinations ZRG-79 × PAK-13 high GCA are best suited for hybrid development.

## Abstract (ru)

Пшеница (Triticum aestivum L.) – основной пищевой и кормовой продукт на территории Пакистана. Одной из приоритетных задач является работа по улучшению сортов пшеницы, отличающихся более высокой урожайностью зерна и устойчивостью к заболеваниям. Ключевым фактором такой работы стало изучение генетического разнообразия сортового материала. С этой целью были оценены показатели урожайности у потомства, полученного от скрещивания четырех элитных сортов пшеницы с тремя тестерными линиями. На основе проведенного анализа получены данные о генетической дисперсии, общей и специфической комбинационной способности, гетерозисе и степени устойчивости к желтой ржавчине (Puccinia striiformis L.). Высокая урожайность зерна (3358 кг/га) была отмечена среди растений первого поколения от скрещивания ZRG- 79 × PAK-13. Дисперсионный анализ (ANOVA) выявил статистически достоверную генотипическую дисперсию по данному признаку. Значение показателя наследуемости (H2) фиксировалось в диапазоне от 28 до 100 %. Выявленная общая комбинационная способность (ОКС) по признаку «урожайность зерна» была статистически достоверной для всех родительских cортов, кроме FSD-08 и PS-05, в то время как специфическая комбинационная способность (СКС) по данному признаку оказалась высокодостоверной только для гибридных растений от двух скрещиваний: ZRG- 79 × NR-09 и ZRG-79 × PAK-13. Величина гетерозиса составила от –28 до 62.6 %. В комбинациях скрещивания ZRG-79 × PAK-13 была показана высокодостоверная величина гетерозиса (62.6 %). Наблюдалась высокодостоверная корреляция по признакам «длина колоса», «число колосков в колосе», «высота растения» и «масса 1000 зерен». Значение индекса устойчивости к ржавчине изменялось в диапазоне от 0 до 8.5. На основании полученных результатов сделаны следующие выводы: 1) учет ОКС важен при отборе на более высокую урожайность зерна, обусловленную действием аддитивных генов; 2) отбором в дочерних поколениях обеспечится повышенная устойчивость к ржавчине; 3) комбинации скрещивания ZRG-79 × PAK 13 с высокой ОКС лучше всего подойдут для создания гибридных сортов.

## Introduction

Wheat (Triticum aestivum L.) is an important cereal crop worldwide playing a crucial role in the daily dietary and nutritional requirement not only for human beings but also for animals. It is the major food for one third of world population and its chief use is the flour for making bread. It is grown around all continents. Increasing human population, climate change and global pandemics have an overwhelming impact on food security, especially wheat on crop with current inadequate genetic improvement of wheat to meet future demand. In Pakistan, wheat is grown in an area of 9.2 million ha with the production of around 25.5 m tonnes (FAOSTAT, 2016) and hardly meets the total requirement of the country. But this figure is continuously under fluctuation because of stagnant yield of cultivars, disease impact, drought, and floods. Apart from these factors, injudicious selection of parental selection for a breeding program without prior knowledge of genetic behaviour in germplasm and lack of indigenous breeding programs for genetic improvement of wheat is another constraint in the yield.

Genetic recombination in germplasm by hybridization is a robust conventional breeding tool for obtaining transgressive segregants and genetic variation, which provides means of selection of ideotypes. Gene action and combining ability analysis are a most reliable biometric procedure for the study of genetic behaviour of yield and yield-related components (Rashid et al., 2007). General combining ability is the average performance of genotypes in a series of cross combinations, while specific combining ability is the performance of a particular genotype in a specific cross combination. Mode of selection depends based on genetic action in traits of interest (Arzu, 2017).

In self-pollinated crops, especially in wheat, plant breeders are usually interested in selection of segregants having additive gene action with high specific combining ability. Additive gene action boosts yield and yield components by cumulative addition of genes. Dominance genetic variance exploits heterosis in cross combinations and specific combining ability provides the presence of dominant or non-additive gene action in a particular trait (Kaushik, 2019) and provides optimal parental identification (Fakthongphan et al., 2016). Equal magnitude of both general and specific combining ability in a breeding population means preponderance of both additive and dominant genes for the traits of interest; selection in this case is most effective for variety development (Ahmad et al., 2012). The term combining ability was first introduced and further refined as general combining ability (GCA) and specific combining ability (SCA) by Sprague and Tatum (1942). GCA distinguishes between the mean performances of parents in cross combinations whiles SCA is the deviation of individual crosses from the average performance of the parents involved. GCA and SCA represent the additive and nonadditive portions of genotypic variance respectively (Hallauer et al., 1988). The estimates from GCA and SCA provide an assessment of relative merits of the individual genotypes in cross combinations to guide selection and testing schemes. Thus, line × tester analysis is among the genetic-statistical approaches developed to assist in selection of parents based on their combining ability and the potential to produce promising segregating populations (Okello et al., 2006). According to GCA and SCA impacts, positive values are desirable for most crop plants characteristics, such as growth and yield-related attributes. Negative GCA and SCA impacts, on the other hand, are desirable for characters where minimum values are essential and appealing, such as early flowering.

Heterosis is a phenomenon where F1 hybrids are superior in traits as compared to their parental genotypes. There are several theories that explain the genetic basis of heterosis, including over-dominance, dominance, and genetic balance. The over-dominance theory of heterosis, first proposed by Shull and East (1908), suggests that heterozygous individuals, since they carry two different alleles, have an advantage over homozygous individuals as they carry two identical alleles for a particular gene. This advantage is thought to mean that the two different alleles can supplement with each other, leading to a vigorous phenotype in F1 hybrids. The dominance theory, presented by Jones (1917), suggests that hybrid vigour is caused by dominant alleles that are more valuable than the recessive alleles. According to this theory, F1 hybrids accede two copies of the dominant allele, resulting in a vigorous phenotype. The third heterosis theory is the “Lerner’s genetic balance theory”, suggested by Lerner (1954), that describes that heterosis is the result of a balance between the expression of genes that promote growth and those that hamper growth. In F1 hybrids, the expression of growth-promoting genes is increased, whereas the expression of growth-retarding genes is decreased, leading to better growth and development.

Heterotic studies for increasing wheat grain yield have been an interest of early wheat researchers. Mid-parent heterosis is the percent of the increase or decrease in the F1 value as compared to the average value of both parents for any metric trait. In the early green revolution era Pal and Alam (1938) reported mid-parent heterosis (MPH) in wheat. After the green revolution and introduction of semi-dwarf wheat varieties, various wheat researchers reported MPH heterosis in wheat (i. e., Knott, 1965; Shamsuddin, 1985; Uddin et al., 1992). Barbosa-Neto et al. (1996) reported MPH in soft red winter wheat in the range of –20 to 57 %. Liu et al. (1999), Dreisigacker et al. (2005), Basnet et al. (2019) reported MPH in CIMMYT wheat varieties in the range of 9.5 to 14 %.

Wheat crop faces numerous challenges that cause yield losses, including stripe rust (Puccinia striiformis f. sp. tritici), which is a major disease in areas where cool to mild warm temperature prevails during the months of February and March in the wheat-growing season. Under conducive environmental conditions, disease causes yield losses ranging from 10 to 70 % depending upon susceptibility of genotypes (Raza et al., 2018). Development of cultivars containing genetic resistance is the most cost-effective and environmentally friendly strategy to mitigate yield losses by stripe rust (Ali Y. et al., 2020). Stripe rust spores continue to mutate and evolve new virulent races causing damage to previously resistant cultivars (Chen et al., 2010). Wheat crop in Pakistan has faced severe damage caused by stripe rust pathogen in recent years (Ali Y. et al., 2020). Due to climate change and rapid mutation in stripe rust pathogen, new races overwintering on alternative host barberry in hilly areas at high altitudes evolve (Figueroa et al., 2020). Under these circumstances, the already resistant genotypes become susceptible (Javaid et al., 2018).

There are two types of resistance mechanism against rust pathogens in wheat, vertical resistance, and horizontal resistance. Vertical resistance is conferred by a single gene to a specific pathogenic race of rust, while horizontal resistance involves the use of multiple genes that provide broad spectrum disease resistance against multiple pathogenic races of rust. There are several resistance genes present in the Pakistani bread wheat varieties that confer resistance against yellow rust, which include Yr5, Yr10, Yr15, Yr17, and Yr18. Qamar et al. (2014) reported Lr34/Yr18 gene complex that confers broad spectrum resistance against yellow rust and leaf rust in most of Pakistani wheat varieties. Intikhab et al. (2021) reported the presence of Lr46/Yr29 gene complex in Punjab-2011 and Pirsabak-2005 cultivars that confer resistance against stripe rust. Khan S.N. et al. (2022) reported the presence of Yr17 and Yr5 gene complex in Pakistani wheat varieties Punjab-2011 and Pirsabak-2005. Utilization of these resistance sources in the breeding program for development of varieties resistant against stripe rust is an ultimate objective to ensure high yield on sustainable basis.

Various biometrical techniques and breeding designs are used for genetic evaluation and genetic behaviour of germplasm to be utilized in crop breeding programs, but line × tester analysis is an efficient mating design providing reliable information about GCA and SCA that ultimately depicts the mode of gene action in a particular trait (Fellahi et al., 2013). GCA and SCA are important to apprehend the genetic architecture of quantitative traits and create the road map for initiation of an efficient breeding program (Fasahat et al., 2016).

Several studies investigating the GCA and SCA effects have been conducted in wheat. Zhao et al. (2013) reported significant effects for both GCA and SCA for yield and its components and inferred that selecting parental genotypes with high GCA and SCA effects could lead to the development of high-yielding wheat hybrids. Similarly, researchers assessed the GCA and SCA effects in spring wheat and durum wheat F1 hybrids by using line × tester model for combining ability estimate and concluded that GCA effects were more important than SCA effects for grain yield and yield-related traits, and selection of parental genotypes with high GCA effects could increase the prospective yield of wheat hybrids (Iqbal A. et al., 2017; Ishaq et al., 2018; Dragov, 2022). They found that both GCA and SCA effects were significant for grain yield and its components and suggested that selecting parents with high GCA and SCA effects could lead to the development of high-yielding wheat hybrids. Selecting parents with high GCA and SCA effects can improve the yield potential and disease resistance of wheat hybrids, and the use of line × tester designs can provide valuable information about the genetic effects of parents and their hybrids

The objectives of this study is to elucidate the general and specific combing ability, heterotic potential, and stripe rust (Puccinia striiformis f. sp. tritici) resistance behaviour of indigenous elite wheat varieties and their breeding population.

## Materials and methods

Experimental site and plant material. The research was carried out at the experimental site of a wheat research program, National Agricultural Research Center, Islamabad Pakistan (Latitude: 33.71° N, Longitude: 73.06° E, Elevation: 683 m) during 2017–2018 wheat growing season. The soil type of the site is clay loam from 0 to 20 cm, and at the 20–40 cm depth it is moderate clay loam. Five widely adopted approved wheat varieties were used as lines (Faisalabad-2008, Punjab-2011, Pirsabak-2005, Miraj-2008 and Zargoon-79) and three widely adopted, registered and approved varieties for rainfed areas of Pakistan were used as a tester, namely, NARC-2009, Pakistan-2013 and Borlaug-2016 (Table 1). These testers are widely adopted and due to their ability to withstand rainfed and drought-prone areas of Pakistan their leaves have the ability to stay green during high terminal heat and drought stress.

**Table 1.**
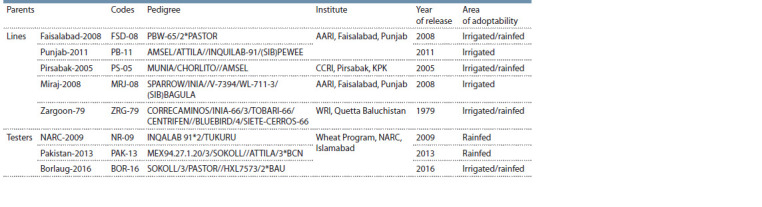
Details of wheat parents used in the study and their pedigree/parentage, year of release and parental Institute

Field experiment and crossing scheme. Eight parents were hybridized to produced 15 F1 cross combinations according to line × tester crossing fashion as described by Kempthorne (1957) during 2017–2018 wheat growing season and crossing was conducted during March 2018. 15 cross combinations and seven parents were planted in Randomized Complete Block Design (RCBD) with three replications during 2018– 2019 wheat growing season. In every replication, parents and F1 hybrids were sown in 1 m length with row-to-row spacing 25 cm and plant-to-plant spacing 15 cm. The experiment was conducted in an irrigated field and a total of 6 irrigations were applied after sowing to harvesting time. Recommended doses of fertilizers, i. e. 120 kg N · ha–1 and 80 kg P · ha–1, were applied. Half of the fertilizers were used at the time of soil preparation, the second half was applied at the time of tillering, and weedicides (Ally Max™ Syngenta and Axial™ Syngenta) were used for eradication of broad leaves and narrow leaves weeds respectively according to the doses mentioned by the manufacturer. Herbicide was applied before the jointing stage of the crop. Leaf area was measured when leaves were fully turgid and green.

Data collection. Grain yield and some yield-related parameters were measured in parents and hybrid combinations. Grain yield per plant was measured in grams and 1000-grain weight was measured after counting 500 grains of each wheat grain sample on a counting tray once and the second sample was repeated for the other 500 grains.

Canopy temperature was measured by using a portable thermal gun (Model: AG-42, Telatemp Crop, CA). Readings for canopy temperature were taken at three Feeks stages (Large, 1954) like booting, kernel water ripening and grain milking stages (Feeks 10, 10.5.4 and 11.2). All readings were taken at the angle of 30° and above 50 cm of the crop canopy, avoiding land temperature by pointing thermal gum only at the canopy. The observations were taken between 11:00 am and 14:00 pm under stagnant air conditions and clear sky as described by (Reynolds et al., 1998). Observations for Normalized difference vegetative index (NDVI) were recorded 50 cm above the canopy by using a hand-held Green Seeker with an optical sensor unit (Model: 505, CA, USA) at three stages of booting and grain filling between 11:00 hours to 14:00 hours with clear sky (Sultana et al., 2014). Values of NDVI range from –1 (NDVI value usually in the water) to +1 (the strongest green vegetative stage) (Kumar, Silva, 1973).

Statistical analysis. Data for other traits (days to 50 % heading, plant height, number of tillers per plant, peduncle length, spike length, days to maturity, number of spikelets per spike, number of grains per spike) were recorded from 6 randomly selected plants. Data recorded were arranged in mean data and subjected to Analysis of Variance (ANOVA) according to Steel and Torrie (1980) and Line × Tester analysis, according to Kempthorne (1957), combining ability and gene action were studied (Singh R.K., Chaudhary, 1977) by using R Package agricolae (De Mendiburu, Simon, 2015; The R Project…, 2017). Genotypic variance and phenotypic variance were estimated as mentioned by Almutairi (2022) in MS Excel 2016, by using the following formula:

**formula. 1.**
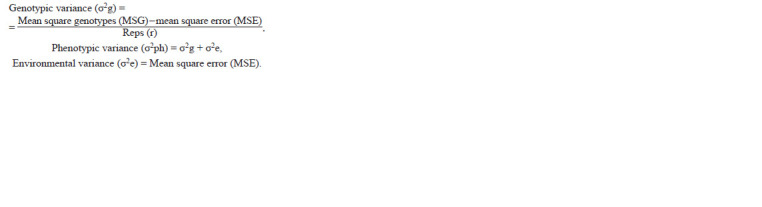
formula. 1.

Environmental variance was estimated according to Comstock and Robinson (1952). Broad sense heritability was calculated by using the following formula as described by Burton and Devane (1953):

**formula. 2.**
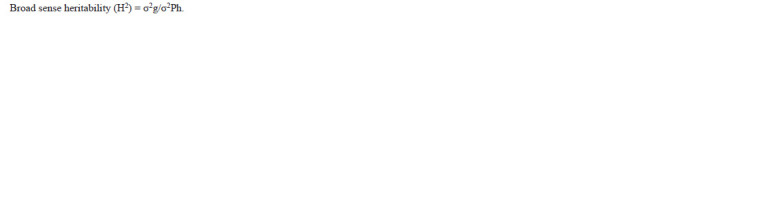
formula. 2.

Heterosis was estimated in percentage increase or decrease of the F1 hybrids value over mid-parental value by following the formula as described by Fonseca and Patterson (1968):

**formula. 3.**
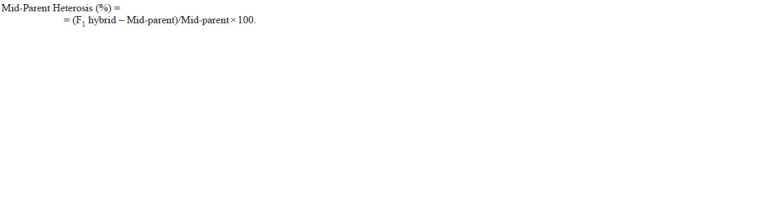
formula. 3.

Disease observations and scoring. Observations for stripe rust were recorded at the time of appearance of disease and data were recorded when rust pathogen was fully developed on leaves of a susceptible check cultivar and leaves’ surface was fully covered with rust’s spores. Disease observation was recorded in three replicates of each parental line and F1 hybrids according to the Cobb Scale method as described by Peterson et al. (1948). The severity of disease was expressed as the percentage of leaf area covered, and 0 % score was given when there was no infection on the leaf and 100 % score was considered when the leaf area was fully covered with rust spores and infection. Readings of percent severity were recorded with the following descriptions for scoring and response values: (R, resistant = 0.2; S, susceptible = 0.3; MR, moderately resistant = 0.4; MRMS, moderately resistant to moderately susceptible = 0.6; MS, moderately susceptible = 0.8; MSS, moderately susceptible to susceptible = 0.9; S, Susceptible = 1.0), response values, coefficient of infection (CI), average coefficient of infection (ACI), country average relative percentage attack (CARPA) and rust resistance index (RRI) according to Akhtar et al. (2002). The following formula was used for the calculation of RRI:

**formula. 4.**
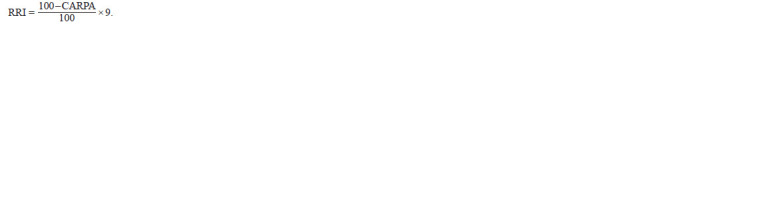
formula. 4.

RRI was calculated by considering the scale of 0 to 9 from CARPA, where 0 represents a most susceptible genotype and 9 represents a highly resistant response of the genotype to rust pathogen.

## Results and discussion

Analysis of variance (ANOVA)

Analysis of variance (ANOVA) results presented in Table 1 show that the lines (female) had statistically significant differences for all the trails. The testers (male) showed statistical differences for days to heading, plant height, peduncle length, spike length, days to maturity, grains per spike, 1000-grain weight and grain yield, while non-significant results for flag leaf area, tillers per plant and spikelets per spike were shown. Interaction of line × tester was significant in case of plant height, flag leaf area, peduncle length, days to maturity, grains per spike and 1000-grain weight. Parents (male and female) used in this study provided a broad range of expression for various characters as shown in Table 2. There were significant differences ( p ≤ 0.05) among the means of genotypes (Table 3) for days to heading (DH), highly significant ( p ≤ 0.01) for plant height (PH), flag leaf area (FLA), tillers per plant (TPP), peduncle length (PL), spikelets per spike (SPS), days to maturity (DM), grains per spike (GPS), thousand grain weight (TGW), grain yield (GY) and NDVI value.

**Table 2.**
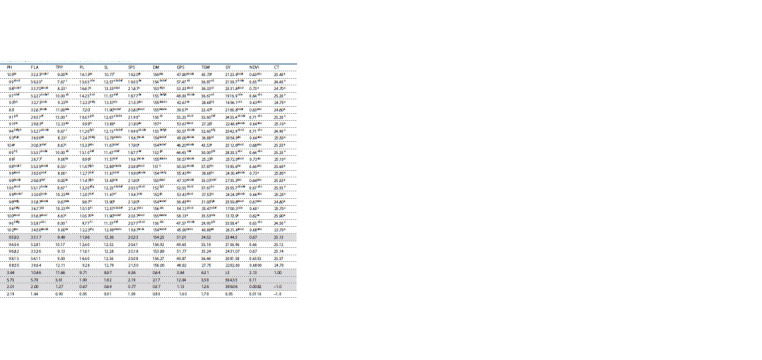
Mean performance of lines, testers, and their cross combinations under rainfed conditions Note. Hereinafter: DH, days to heading; PH, plant height, cm; FLA, flag leaf area, cm2; TPP, tillers per plant; PL, peduncle length, cm; SL, spike length, cm; SPS, spikelets per spike; DM, days to maturity; GPS, grains per spike; TGW, 1000-grain weight; GY, grain yield per plant, kg · ha–1; NDVI, normalized difference in vegetative index, and CT, canopy temperature, °C.

**Table 3.**
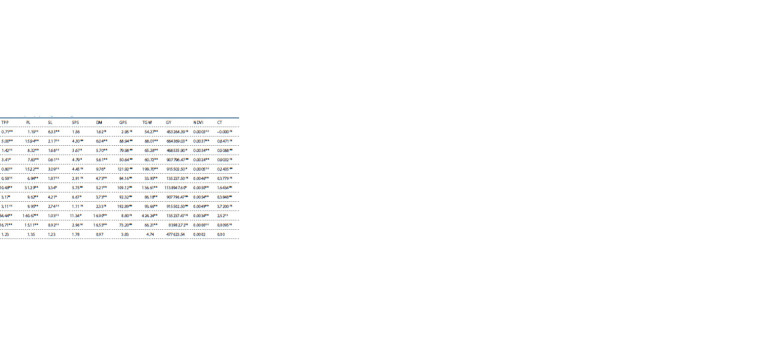
ANOVA for line × tester (including parents) mean squares for morpho-physiological and agronomic traits under rainfed conditions Note. SOV, source of variation; DF, degree of freedom. * Significant at p <0.05, ** significant at p < 0.01, ns significant at p > 0.05.

The values for days to heading (DH) were maximum in the tester (male) PAK-13 (119 days) and minimum in the lines (female) PB-11, PS-05 and MRJ-08 (117 days). DH are the key indicator of earliness in crop production. Plant breeders are keen to create new varieties of wheat genotypes with early maturity. So, early heading is a desirable trait. Delayed heading leads to a reduction in yield (Ullah et al., 2018) and early heading increases the grain filling duration, which ultimately results in high yield (Iqbal A. et al., 2017). Plant height (PH) was the highest in the female parent FSD-08 (103 cm) and the lowest in the male parent NR-09 (83 cm). Minimum PH is preferred due to expected lodging losses. Similarly, the tester NR-09 (83 cm) can be assessed for developing drought tolerant variety with reduced plant height for future breeding programs. Likewise, minimum flag leaf area (FLA) is also desirable for drought tolerance due to reduced transpiration losses from a reduced area exposed to sunlight. The testers (male) PAK-13 and BOR-16 showed the minimum values of flag leaf area: 29.57 and 29.83 cm2, respectively. Peduncle length was longest in the female parent PS-05 (16.67 cm) and shortest in the male parent NR-09 (7.20 cm). PS-05 produced the maximum grain yield (2531.8 kg · ha–1) while minimum grain yield (1696.1 kg · ha–1) was recorded in ZRG-79.

Mean performance of parents and their cross combinations Mean performance for line, testers and cross combinations for days to maturity (DH) ranged from 117 to 119 days. The parental lines FSD-08, PB-11, PS-05, MRJ-08, ZRG-79 and BOR-16 were revealed to have 117 DH, while NR-09 and PAK-13 had 118 and 119 days to heading, respectively (see Table 2). Among F1 hybrids Zargoon-79 × Pakistan-2013 had 119 days for heading while the rest of the cross combinations showed 117 DH. The grand means for parents, crosses, lines and testers were 117.67, 117.56, 117.27 and 118.33, respectively. The coefficient of variance 0.67 % obtained for DH was also in the acceptable range.

Average minimum plant height was recorded in NARC- 2009 (83 cm) followed by cross combination of Punjab-2011 × Pakistan-2013 (86 cm), and maximum plant height of 104 cm was recorded in the cross-combination Faisalabad-2008 × Borlaug-2016 followed by one of parent viz. Faisalabad-2008 (103 cm). Grand mean, coefficient of variance (CV) and least significant variance (LSD) for plant height of lines, testers and their parental combinations was revealed to be 95.92 cm, 3.64 and 5.73, respectively.

The cross-combination Punjab-2011 × Pakistan-2013 showed minimum value (26.8 cm2) for flag leaf area followed by the lines Pakistan-2013 (29.5 cm2) and Borlaug-2016 (29.8 cm2). Maximum leaf area was recorded in the line Punjab- 2011 (39.2 cm2) followed by the F1 combination, Faisalabad- 2008 × Pakistan-2013 (36.9 cm2). Grand mean, CV, LSD and standard error for leaf area of lines, testers and cross combinations was recorded as 10.46, 5.7 and 2.0 cm2 respectively.

Maximum 13 tillers per plant (TPP) was recorded in the tester Pakistan-2013 followed by 12.3 tillers in Borlaug-2016 while minimum 7.6 tillers were observed in the female parent Punjab-2011 followed by Pirsabak-2005 (8.33). In the cross combinations a maximum of 10 tillers was recorded in Punjab-2011 × NARC-2009 and Miraj-2008 × NARC-2009 and grand mean for TTP was recorded as 9.49 with CV, LSD and SE 11.66, 3.61 and 1.27 respectively.

Maximum peduncle length was observed in the line Pirsabak- 2005 while minimum peduncle length was recorded in the tester parent NARC-2009. Grand mean for peduncle length was observed to be 11.96 cm with CV 9.7 % and LSD (α 0.05) value 1.9.

Mean performance for spike length (SL) was observed 12.36 cm in parents and their cross combinations. Maximum SL was observed in cross combinations Miraj-2008 × Pakistan-2013 followed by Miraj-2008 × Borlaug-2016, but minimum SL was observed in the parental line Faisalabad- 2008.

Grand mean for spikelets per spike (SPS) for parents and cross combination was recorded as 20.33 with a maximum of 21.9 spikelets observed in Pakistan-2013 in addition to the F1 hybrid combination of Miraj-2008 × Pakistan-2013 and in the Pirsabak-2005 × Pakistan-2013. Grains per spike (GPS) was recorded maximum in parental line Punjab-2011. Average thousand grain weight was calculated to be 34.52 with higher TGW in Faisalabad-2008 (43.73 g) and the cross combination of Faisalabad-2008 × Pakistan-2013 (43.53 g), and lower value for TGW was depicted by the parental line NARC-2009 (22.47 g). Average grain yield was obtained in all the parents and cross combinations (2344.5 kg · ha–1), while average GY was higher in crosses as compared to the parents’ grain yield, the maximum was recorded in the cross combination ZRG- 79 × PAK-13 (3358 kg · ha–1), followed by PB-11 × NR-09 (2820 kg · ha–1), while minimum grain yield was recorded in the cross combination of ZRG-79 × NR-09 (1372 kg · ha–1).

Maximum normalized differences in vegetative index (NDVI) value was observed in the parental line PS-05 (0.73) followed by PB-11 × PAK-13 and PS-05 × NR-09 with the same value. Average NDVI value for parents and crosses was revealed to be 0.67; lines, testers and crosses also contained similar values for NDVI.

There were significant differences among the means of crosses combinations for almost all the traits studies except DH, TPP an SL. The lines (female parents) also depicted highly significant differences in all the parameters under consideration except SL. The testers (male parents) also revealed highly significant differences for all the traits except for FLA, SL, SPS, and NDVI. Interaction of lines × testers depicted highly significant differences in their mean performance for the traits of PH, FLA, PL, DM, GPS, TGW and NDVI value.

Estimates of genetic variance components

Estimation of genotypic variance, phenotypic variance, environmental variance, variance due to general combining ability, variance due to specific combining ability and variance due to GCA over SCA is mentioned in Table 4.

**Table 4.**
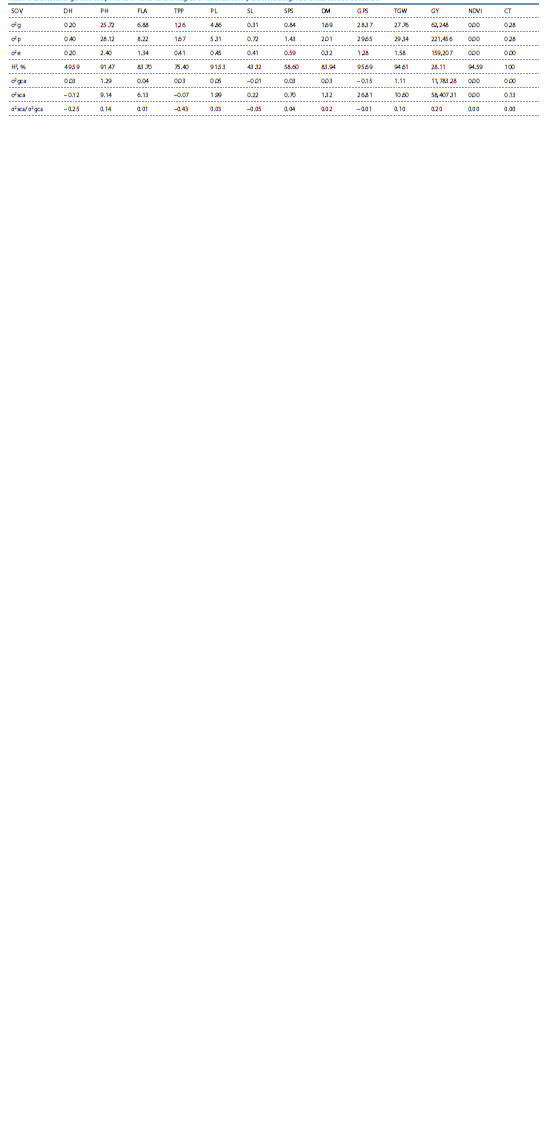
Estimates of genetic components of variance and degree of dominance of yield attributing traits under rainfed conditions Note. σ2g, genotypic variance; σ2p, phenotypic variance; σ2e, environmental variance; H2, broad sense heritability; σ2gca, general combining ability variance; σ2sca, specific combining ability variance.

Phenotypic variance was depicted more as compared to genotypic variance in some traits, i. e. PH, FLA, SL, DM, and GY, while only GY showed high environmental variance. Broad sense heritability (H2) was estimated in the range of 28.11 % (GY) to 100 % (CT). PH, PL, GPS, TGW, NDVI value and CT showed broad sense heritability of more than 91 %, while DH and GY depicted less heritability. The traits with high genetic variance, low environmental variance and high broad sense heritability have preponderance of additive genes and these are stable characters and selection in the filial generations can be made by keeping eye on these traits. Grain yield (GY) and DH attained low broad sense heritability and showed that environmental influence is more important for the expression of these traits. Selection in the filial generation should be made for these traits by considering disease incidence and drought proxy parameters, i. e. NDVI and CT values

Proportional contribution of lines, testers, and their interactions to total variance

**Fig. 1.**
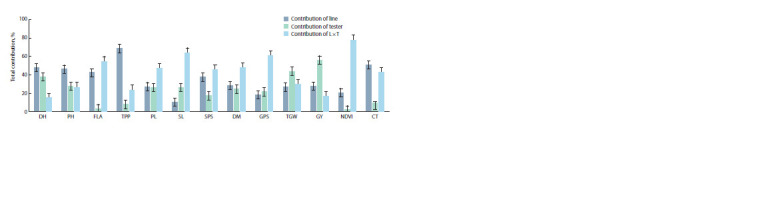
Proportional contribution of lines, testers, and their interactions to total variance under rainfed conditions. Here and in Figure 3: DH, days to heading; PH, plant height, cm; FLA, flag leaf area, cm2; TPP, tillers per plant; PL, peduncle length, cm; SL, spike lenth, cm; SPS, spikelets per spike; DM, days to maturity; GPS, grains per spike; TGW, 1000-grain weight; GY, grain yield per plant, kg · ha–1; NDVI, normalized difference in vegetative index, and CT, canopy temperature, °C.

Proportional contribution of total variance for yield and yield-related metric traits for lines, testers and their cross combinations was estimated (Fig. 1). For DH, PH, TPP and CT it was recorded to be higher as compared to the testers and combinations of both lines and testers. Contribution of L×T to total variance was recorded as high in FLA, PL, SL, SPS, DM, GPS and NDVI value, while variance contribution of testers to TGW and GY was estimated higher as compared to the lines and L×T combinations.

General combining ability

General combining ability (GCA) estimates for all the traits are given in Table 5. Both positive and negative GCA effects were observed for lines and testers. For DH, the value of GCA effects ranged between 0.00 and 0.56. As a good general combiner, significant positive (0.56) and negative (–0.56) GCA effects were observed for lines Zargoon-79 and Pirsabak-2005, respectively. Similarly, in the testers, positive and significant (0.51) GCA effect was observed for Pakistan-2013 only (see Table 5). Patel et al. (2020) demonstrated ( p ≤ 0.01) significant negative and desirable GCA effects in lines and non-additive gene action was primarily involved in days to heading.

**Table 5.**
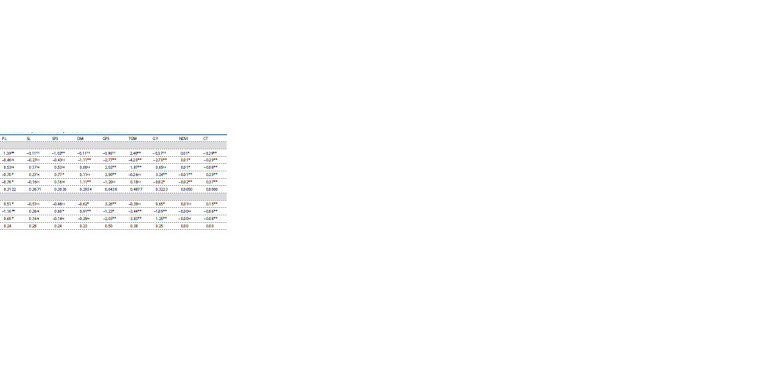
General combining ability effects of wheat genotypes, lines and testers for yield and its components under rainfed conditions

For plant height, negative general combining ability effects are more important since more emphasis is placed upon selection for short stature in segregating the population because it ultimately turns out that a short stature line is more responsive to fertilizer and tolerant to lodging. In this study, GCA effects ranged between –5.82 and 3.24 for PH. Significant positive (3.07) and negative (–5.82) GCA effects were observed for the lines Pirsabak-2005 and Punjab-2011, respectively. Similarly, highly significant positive (3.24) was estimated for the tester BOR-16 and highly significant but negative (–2.56) GCA effects were observed for the testers PAK-13, respectively. These results are in accordance with the results of (Singh S. et al., 2003; Gorjanović, Kraljević-Balalić, 2007).

For flag leaf area (FLA), negative general combining ability effects are more important because FLA is much influenced by transpiration losses due to disclosure to sunlight, which eventually affects the grain yield. Hence, more emphasis is retained on the selection of genotypes with smaller FLA. From that, among the female parents, Pirsabak-2005 and Punjab-2011 showed a highly significant negative GCA effect: –2.16 and –2.10, respectively. On the other side, no significant GCA effects were observed among the testers for FLA. These results confirm the findings of (Saeed A. et al., 2001; Arshad, Chowdhry, 2002; Chowdhary et al., 2007).

In case of tillers per plant (TPP), GCA effects ranged between –0.58 and 0.98. As a good general combiner, highly significant positive (0.98) GCA effects were observed only for the line MRJ-08 while there were no significant GCA effects among the testers for TPP. To begin with, TPP is a significant yield-boosting characteristic that contributes to increased grain yield. A higher number of tillers per plant confirms optimal plant populations and as a result higher grain yield (Tilley et al., 2019). For this point of view, the female line MRJ-08 showed better performance. These findings are in accordance with the results of (Iqbal M.M., 2007; Khan A. et al., 2020; Rashmi et al., 2020).

GCA effects ranging between –1.16 and 1.39 were observed for peduncle length (PL). Highly significant positive (1.39) and negative (–0.76) GCA effects were observed for the lines (female) FSD-08 and ZRG-09, respectively. In the same way, highly significant positive (0.65) and negative (–1.16) GCA effects were observed for the testers BOR-16 and PAK-13, respectively. Likewise, in PH, shorter PL is preferred because an increase in PL ultimately increases the PH and we prefer a plant with short stature. In current study, two female parents, ZRG-79 (–0.76) and MRJ-08 (–0.70), showed negative general combining ability. Also, one male parent, PAK-13, showed superior general combining ability for this trait. So, it can be concluded that the above-mentioned parents are desirable for use in the breeding program. The findings of (Sharma, Garg, 2005) supported the results.

Greater spike length (SL) and larger number of spikelets per spike (SPS) are essential for enhanced yield. Among parents, one line (female), MRJ-08, showed significant positive values (0.77) for SPS. One tester, Pakistan-2013, exhibited high GCA for SPS. These results were quite close to the findings of (Awan et al., 2005; Sharma, Garg, 2005; Hassan et al., 2007). Number of grains per spike (GPS) is also an important factor for enhanced grain yield. Therefore, positive GCA effects are more important due to positive contribution of grain yield. Among male parents, only NR-09 showed positive and higher values (3.26) of GCA effects for GPS. Among female parents, MRJ-08 and PS-05 showed positive and higher values, i. e. 2.90 and 2.02 respectively. It should be noted that values of male parents were higher than those of female parents. These findings match with the results of (Saeed A. et al., 2001; Ahmadi et al., 2003; Saeed M.S. et al., 2005; Hassan et al., 2007). These results are different from the findings of Nazir et al. (2005).

For grain yield per plant (GY), only one female parent MRJ-08, and among the male parents, BOR-16 and NR-09, exhibited positive general combining ability effects. Similar results were also found by (Malik et al., 2005).

Specific combining ability

Specific combining ability (SCA) estimates for all the traits are given in Table 6. Both positive and negative SCA effects were observed among the crosses.

**Table 6.**
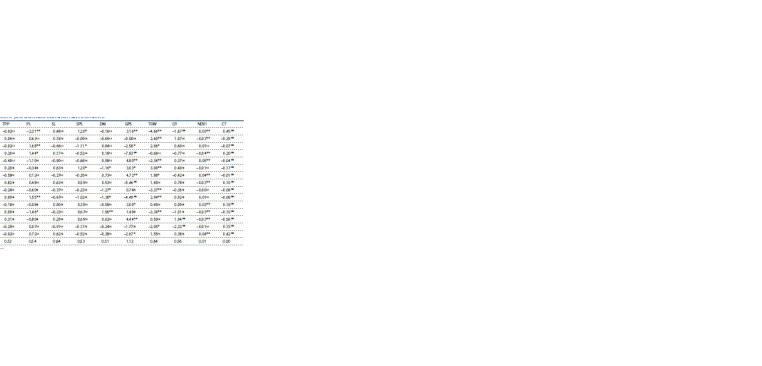
6. Specific combining ability effects of 15 wheat crosses for yield and related traits under rainfed conditions * Significant at p < 0.05; ** significant at p <0.01; ns significant at p > 0.05.

As for SCA effects for DH, all the fifteen crosses were of non-significant nature with positive and negative magnitude (see Table 6). The result indicates the involvement of both additive and non-additive genetic effects in the inheritance of DH, with greater proportion of additive genetic effect. Lines with maximum SCA effects can be used in development of hybrid cultivars. Only six among fifteen crosses depicted negative SCA effects for plant height. If parents with tallness are the ideal ones, then the crosses FSD-08 × NR-09, FSD-08 × PAK- 13, PB-11 × NR-09, PB-11 × PAK-13, PS-05 × BOR-16 and MRJ-08 × BOR-16 would be considered good. However, the remaining crosses exhibited higher SCA effects. These findings confirmed the results of (Arshad, Chowdhry, 2002; Hasnain et al., 2006; Chowdhary et al., 2007). Furthermore, non-additive type of gene action is detected for PH and supported by (Babar et al., 2022). Also, our results concur with Ali F.K.H. and Abdulkhaleq (2019) for plant height.

GCA effects for flag leaf area range from negative –3.80 to positive 3.33. Roughly 50 % of the crosses showed smaller values of SCA effects for flag leaf area, which is desirable. As less flag leaf area is required for drought tolerance, the crosses with significant SCA effects, i. e. FSD-08 × BOR-16 and PB-11 × PAK-13 may be used in a future breeding program because they have high negative SCA values contributing towards minimum FLA. However, the remaining crosses exhibited higher positive SCA effects for FLA. Comparable results have also been stated by (Saeed A. et al., 2001; Arshad, Chowdhry, 2002; Chowdhary et al., 2007).

Negative SCA effects are needed to reduce the peduncle length (PL). In this study, two crosses showed significantly negative SCA effects. FSD-08 × NR-09 and MRJ-08 × BOR- 16 are the best hybrids for reduced PL. Similar results were reported by (Chowdhary et al., 2007).

In case of spike length (SL), all the fifteen crosses were of non-significant nature with positive and negative magnitude (see Table 6). For a number of SPS, positive specific combining ability effects were shown in 6 out of 15 crosses but only two crosses, FSD-08 × NR-09 and PB-11 × BOR-16, have significant GCA effects. These hybrids performed best and can be suggested for future breeding programs. These results are in the conformity with those of (Mahantashivayogayya et al., 2004).

For grain yield per plant, SCA effects found varied much among crosses. The poorest cross with respect to SCA for grain yield per plant was ZRG-79 × PAK-13 whereas the cross that appeared to be the best and the most promising specific combination was ZRG-79 × NR-09. Positive specific combining ability effects were displayed in 8 out of 15 crosses. But only ZRG-79 × NR-09 showed such significant positive effects among crosses. Similar results were also reported by (Saeed A. et al., 2001).

Mid-parent heterosis estimation for grain yield

**Fig. 2.**
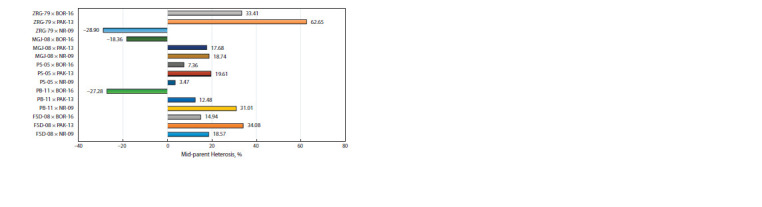
Estimation of mid-parent heterosis for grain yield (GY) as a percentage increase or decrease in the F1 hybrids compared to mid-parental value.

Mid-parental heterosis (MPH) for GY was estimated for 15 F1 hybrids (Fig. 2). F1 hybrids ZRG-79 × PAK-13 showed higher mid-parental value (62 %) followed by FSD-08 × PAK- 13, ZRG-79 × BOR-16, and PB-11 × NR-09, which revealed mid-parent heterosis value above 30 % (34, 33, 31 % respectively). Cross combinations PS- 05 × PAK- 13, MRJ- 08 × NR-09, MRJ-08 × PAK-13, FSD- 08 × NR- 09 depicted mid-parental heterosis value more than 15 %. Three cross combinations, ZRG-79 × NR-09, MRJ-08 × BOR- 16 and PB-11 × BOR-16, depicted negative heterosis.

Cross combinations with more than 30 % mid-parental heterosis can be used in hybrid breeding in wheat. Heterotic studies for increasing wheat grain yield has been an interest of early wheat researchers. Pal and Alam (1938) reported mid-parent heterosis in the pre-green revolution era. After the introduction of semi-dwarf wheat in the post-green revolution era, various wheat researchers reported mid-parent heterosis in wheat, i. e. (Knott, 1965; Shamsuddin, 1985; Uddin et al., 1992). Barbosa-Neto et al. (1996) reported MPH in red soft winter wheat in the range of –20 to 57 %. Liu et al. (1999), Dreisigacker et al. (2005), Basnet et al. (2019) studied MPH in CIMMYT wheat varieties and reported MPH in the range of 9.5 to 14 %. Parental lines and tester used in present studies have CIMMYT background and the majority of the genotypes exhibited similar results for MPH. However, crosses combination ZRG-79 × PAK-13 has one indigenous parent ZRG-79 and exhibited a high percentage of MPH. These finding can demonstrate that crosses among parents with CIMMYT background have low heterotic potential and additive gene action governed the GY potential in these cross combinations and selection in the filial generation will be key for transgressive segregants, but in case of crosses among indigenous parents and genotypes with a CIMMYT parent it will be good source of hybrid breeding.

Correlation study of agronomic traits

**Fig. 3.**
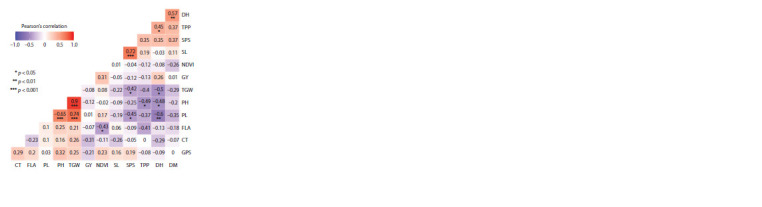
Correlation study among yield and related traits of eight parents and 15 wheat crosses under rainfed conditions.

Correlation study among yield and related traits under rainfed conditions of eight parents and 15 wheat crosses is mentioned in Figure 3. High significance was observed between PH and TGW with the value of 0.9 ( p < 0.001), SL and SPS had a correlation coefficient value of 0.72 ( p <0.001) followed by DH and DM with 0.57 ( p <0.01). PL also showed highly significant and positive correlation with TGW and PH (0.74 and 0.65 respectively, p <0.001). PL and TGW also revealed significant but negative correlation with DH –0.6 and –0.5, respectively ( p < 0.01).

Positive and significant correlation among PH and PL with TGW showed that the higher the plant height the higher the thousand grain weight and peduncle length. Careful consideration should be made while selecting the genotypes with stiff and strong stem girth to avoid lodging. Correlation between SL and SPS revealed that an increase in spike length leads to an increase in spikelets per spike, genotypes with long spikes will be a good selection criterion for increasing yield due to the increase in number of spikelets per spike. Positive and significant correlation among DH and DM depicted that genotypes with early DH would mature earlier, so selection of genotypes with early flowering is good for early maturity and short duration variety development. Significant but negative correlation between TGW and DH indicated that a delay in days to flowering leads to a reduced TGW and vice versa. TGW showed negative correlation with TPP and these findings are in line with the results of Almutairi (2022). Low correlation of GY with other parameters in wheat was also reported by Gowda et al. (2010).

Stripe rust responses of parental lines and their cross combinations

The response to stripe rust (Puccinia striiformis f. sp. tritici) on parental lines used in the study and their offspring (crosses) is recorded for disease scoring, coefficient of infection (CI), average coefficient of infection (ACI), country average relative percentage attack (CARPA) and rust resistance index (RRI) (Table 7). All the parental lines showed moderate resistant (MR) to highly resistant (R) reaction against stripe rust (Pst). The female parents (lines) FSD-08, PB-11, PS-05, MRJ-08 and ZRG-79 showed 20M, 20M, 5M, 30M and 40M scores respectively, while the pollen parents (testers) viz. PAK-13, BOR-16 and NR-09 depicted 10MR, 5R and 40M response against stripe rust. All these parents showed a slow rusting response against rust pathogen that is under the control of multiple genes

**Table 7.**
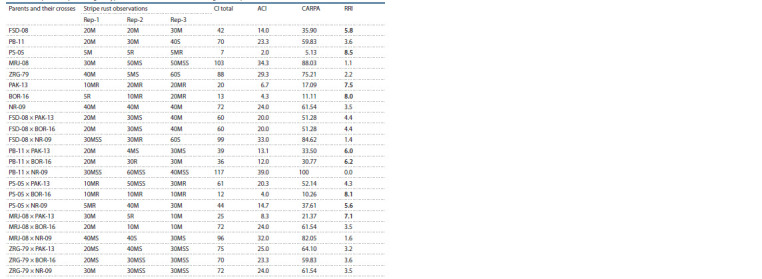
Response of parental genotypes and their cross combinations against stripe rust infection under rainfed conditions Note. R, resistant; S, susceptible; MR, moderately resistant; MS, moderately susceptible; MSS, moderately susceptible to susceptible; CI, coefficient of infection; ACI, average coefficient of infection; CARPA, country average relative percentage attack; RRI, relative rust index.

Cross combinations of these parental lines showed a varied response, moderately resistant to moderately susceptible reaction against stripe rust. The F1 hybrids combinations PS- 05 × PAK-13, PS-05 × BOR-16 and PS-05 × NR-09 showed 10MR, 10MR and 5MR reaction, the crosses FSD-08 × PAK- 13, FSD-08 × BOR-16, PB-11 × PAK-13, PB-11 × BOR-16, and MRJ-08 × BOR-16 showed 20M reaction, the cross combination MRJ-08 × PAK-13 showed 30M reaction, while the rest of the crosses showed moderately susceptible to susceptible reaction against stripe rust.

Average coefficient of infection (ACI) for the parents PS- 05, PAK-13, BOR-16 and FSD-08 was recorded as 2.0, 6.7, 4.3 and 14.0 respectively and these varieties revealed a very good level of resistance against stripe rust. Rust resistance index (RRI) of these parents was also high (ranged 5.8 to 8.5), which indicated a good resistance response of these varieties. Among the cross combinations, PS-05 × BOR-16, MRJ-08 × PAK-13, PB-11 × BOR-16, PB-11 × PAK-13 and PS-05 × NR-09 depicted ACI values of 4.0, 8.3, 12.0, 13.1, and 14.7, respectively. These F1 hybrids had a resistant response to stripe rust. RRI value of the F1 hybrids (PS-05 × BOR-16, MRJ-08 × PAK-13, PB-11 × BOR-16, PB-11 × PAK-13 and PS-05 × NR-09) was higher (ranging from 5.6 to 8.1).

The higher the RRI value and the lower the ACI value means of genotypes with a resistant response to the disease pathogen and under the influence of slow rusting genes, the slower the disease progress and the lesser the yield losses. Genotypes with higher RRI values (>5.0) represent moderately resistant to highly resistant response against rust pathogen. The parental genotypes viz. PS-05, PAK-13 and BOR-16 had higher values for RRI (8.5, 7.5 and 8.0 respectively) showing a highly resistant response against stripe rust pathogen. Cross combinations revealed an intermediate response against stripe rust as compared to parents, especially testes, and resistant genes are under the control of additive gene action. These results indicate that repeated backcross can be a better strategy for accumulation of resistant genes in these cross combinations. Selection in these cross combinations by following backcrosses with recurrent parents is efficient for disease resistance in the filial generations. These results are very much in line with the findings of Afzal et al. (2009) and Mahmoud et al. (2015).

## Conclusion

According to these findings, it can be concluded that higher general combining ability and low broad sense heritability for grain yield suggest the presence of additive genes, and exploitation of general combining ability for high grain yield is important due to presence of additive gene action, and selection in the filial generations and family rows will be effective. For development of heterotic population, it is important to exploit specific combining ability for dominant gene action by crossing indigenous genotypes with exotic germplasm with improved rust resistance, which will be a useful future breeding strategy

## Conflict of interest

The authors declare no conflict of interest.
